# Ferroptosis, a key to unravel the enigma of the FLASH effect?

**DOI:** 10.1259/bjr.20220825

**Published:** 2022-11-16

**Authors:** Nuria Vilaplana-Lopera, Ammar Abu-Halawa, Ellie Walker, Jiyoung Kim, Eui Jung Moon

**Affiliations:** ^1^ Department of Oncology, MRC Oxford Institute for Radiation Oncology, University of Oxford, Oxford, United Kingdom; ^2^ St. Edmund Hall, University of Oxford, Oxford, United Kingdom; ^3^ Somerville College, University of Oxford, Oxford, United Kingdom; ^4^ Department of Radiation Oncology, Gangnam Severance Hospital, Yonsei University College of Medicine, Seoul, South Korea

## Abstract

Ferroptosis is a non-apoptotic form of cell death dependent on iron and lipid peroxides. It has been recently described to have a role on cell death after radiation (RT) through a DNA damage independent mechanism. While the modification of ferroptosis pathways is suggested to enhance radiosensitisation, normal tissue toxicity may limit the combined treatment of RT and ferroptosis inducers. FLASH RT is given at ultra-high dose rates to reduce normal tissue toxicities, which contributes to the RT effect on the tumour. Although several hypotheses including oxygen depletion, reduced ROS, and immune responses are suggested to explain the FLASH effect, the underlying mechanisms of normal tissue sparing effects are still not well understood. Previous studies highlighting the inverse effect of RT dose rates and lipid peroxidation, along with the hypothesis by Spitz et al, suggest that oxygen depletion from the chain reaction of lipid peroxidation and differences in labile pool between normal and tumour tissues may be related to the normal tissue sparing effect of FLASH. Therefore, the role of ferroptosis in ultra-high dose rate FLASH RT needs to be investigated further as it might be the key to increase the therapeutic window of FLASH RT.

## Introduction

Ferroptosis is a non-apoptotic form of programmed cell death that depends on iron and the accumulation of lipid peroxides.^1^ This form of cell death is genetically, biochemically, and morphologically distinct from other types of regulated cell death such as apoptosis and necroptosis. The hallmarks of ferroptosis include 1) the iron-dependent production of reactive oxygen species (ROS) by the Fenton reaction, 2) the accumulation of lipid peroxides, and 3) defective lipid peroxide repair responses. Morphologically, this is manifested in the shrinkage of mitochondria, a reduction in mitochondrial cristae, and the condensation and rupture of the plasma membrane. The uncontrolled oxidation of lipids results in pore formation, cell swelling and membrane damage eventually leading to cell death.^2^

The term “ferroptosis” was first coined a decade ago by Dixon *et al* after observing that the small molecule erastin could induce cell death in *Ras*-mutant tumour cell lines.^1^ Ever since, ferroptosis has been studied in the context of cancer specifically to target drug-resistant, invasive and aggressive tumours. Inducing ferroptosis by either inhibition of the cystine/glutamate transporter, SLC7A11 (*i.e.,* erastin) or glutathione peroxidase 4 (GPX4) (*i.e.,* RSL3) has shown promising results in a variety of cancers. While these ferroptosis inducers are extensively studied in experimental settings, classical tumour treatments including chemotherapy, radiation and immunotherapy also promote ferroptosis.^3,4^

## Radiation-induced ferroptosis

RT is effective in treating many types of cancers by inducing direct DNA damage and through the generation of ROS that result in the inhibition of DNA damage repair. However, both tumour intrinsic mechanisms for suppressing ROS as well as a hypoxic microenvironment reduce the efficacy of RT. While significant efforts are being pursued to target the DNA damage response (DDR) to enhance RT efficacy, there are still limitations in clinical translation due to the lack of biomarkers, acquired resistance, and toxicities from combined treatments. When these disadvantages are taken into consideration, ferroptosis might be a new approach to promote RT-induced cell killing with a DNA damage independent mechanism.

RT results in a variety of cell death including necrosis, apoptosis, mitotic catastrophe, senescence, autophagy, necroptosis and ferroptosis.^5^ Although the exact contribution of each cell death pathway to RT-induced cell killing is not clear, *in vitro* data by Lei *et al* provide an abstract idea using inhibitors of apoptosis, necroptosis and ferroptosis.^4^ In three lung cancer cell lines (H460, A549, and H1299), 6 Gy of RT resulted in 78–93% of cell death, from which ferroptosis was responsible for 14–18% of cell killing, while apoptosis and necroptosis were accounted for (only) 5–10% and 8–10% of cell death, respectively. In RT-treated tumour cells, several hallmarks of ferroptosis have been observed, such as shrunken mitochondria and increased lipid peroxidation, indicating that RT induces ferroptosis. Furthermore, it has recently been shown that the use of ferroptosis inducers or inhibitors in combination with RT alters the efficacy of the treatment.^3,4,6^ Mechanistically, it is still not entirely clear how RT induces ferroptosis. Although not fully understood, the mechanisms behind RT-induced ferroptosis are clinically important as cancer patient responses to RT were found to correlate with ferroptosis induction. Using 4-hydroxynonenal (4-HNE) staining, a marker of lipid peroxidation, it was shown that lipid peroxidation was increased following RT in oesophageal cancer patients. Its expression was also associated with better clinical outcomes and longer disease-free survival of patients.^4^ Ferroptosis is therefore suggested as a therapeutic target for enhancing RT efficacy. In cancer cells, the combination of RT and ferroptosis inducers such as erastin and RLS3 resulted in synergistic increases in lipid peroxidation and tumour cell death. It has been shown that the FDA-approved drugs sulfasalazine and sorafenib both have radiosensitising effects on cancer cells, likely through their actions on SLC7A11. However, clinical applications are still under debate, as increasing sensitivity to RT may also increase off-target effects in adjacent healthy tissue.

## Flash-RT, oxygen depletion, and ROS

FLASH RT delivers RT at rates often exceeding 100 Gy/s compared to conventional methods, which deliver doses at rates of around 0.1 Gy/s.^7^ While the tumour cell killing efficacy appears to be similar for both conventional and FLASH RT, the latter results in lower normal tissue toxicity. Although the underlying mechanisms of the normal tissue sparing effect are not well understood, the hypotheses suggested to explain this “FLASH effect” are 1) oxygen depletion, 2) decreased ROS production, and 3) modified immune responses.

1. At high dose rates, the radiolysis of water accelerates oxygen consumption before the re-diffusion of oxygen through tissues can maintain the microenvironmental oxygenation.^8^ Since tumour tissues are already too hypoxic, the radio-protection effect is more prominent in normal tissues.
2. In addition to direct damage, RT induces indirect DNA damage through ROS, however, increased radical-radical interaction resulted from enhanced ionisation from high dose rates, results in less tissue toxicity.^9^
3. It has been also shown that the immune response to FLASH RT differs from that of conventional RT due to the short RT time, exposing fewer lymphocytes to RT, and resulting in reductions in both chromosomal aberrations and immune system activation.^10^

It has been confirmed in recent studies that instantaneous depletion of oxygen after FLASH RT occurs both *in vitro* and *in vivo* settings.^11,12^ Although oxygen changes measured in these studies did not fully support FLASH-induced radiological hypoxia to promote normal tissue protection, they indicate that it is necessary to determine actual oxygen levels consumed after FLASH RT including those that are used to produce radicals.

While RT-induced ferroptosis has been reported by recent studies, enhanced lipid peroxidation after RT was first reported by Wills and Wilkins in 1967.^13^ Together with the following studies, it was also determined that the production of lipid peroxides is time, dose, and oxygen dependent, and is enhanced by the availability of iron and lipids.^14^ Interestingly, these studies found inverse dose-rate effects on lipid peroxidation, indicating a higher efficiency in lower dose rate RT, which may be the result of the recombination of highly reactive and short-lived free radicals.^14^ Although the dose rates were given over a narrow range and did not reach those observed in FLASH RT, they still suggest the possibility of the FLASH effect on lipid peroxidation and ferroptosis. It is possible that reduced lipid peroxidation produced by FLASH results in less ferroptosis in normal tissues. However, to support similar tumour effect to conventional RT, the hypothesis by Spitz *et al* can be taken into consideration.^15^ Their model suggests that high dose rate of the FLASH leads to rapid oxygen consumption to convert oxygen into free radicals. Oxygen depletion is further intensified by the chain reaction of lipid peroxidation through the Fenton reaction. The beneficial effect of FLASH stems from the larger labile iron pool in tumour cells, which results in the production of more free radicals, and hence more oxidative damage in tumour. In contrast, lower prooxidant burdens and a greater capacity to rapidly remove free radicals protects healthy tissue after ultra-high dose rate RT.^15^ However, these theories are debated since they model cells as pure water and did not consider the effect of radical-radical interactions. Furthermore, the inverse dose rate effect on lipid peroxidation is not considered. Nonetheless, the differences in labile iron between tumour and healthy tissue can be used to target tumour cells through radiation-induced ferroptosis even at high dose rates of FLASH. Therefore, ferroptosis might be a key to unravel mechanisms of FLASH effect of oxygen depletion and reduced ROS production, which results in normal tissue protection.

## Conclusion

**Figure 1.**
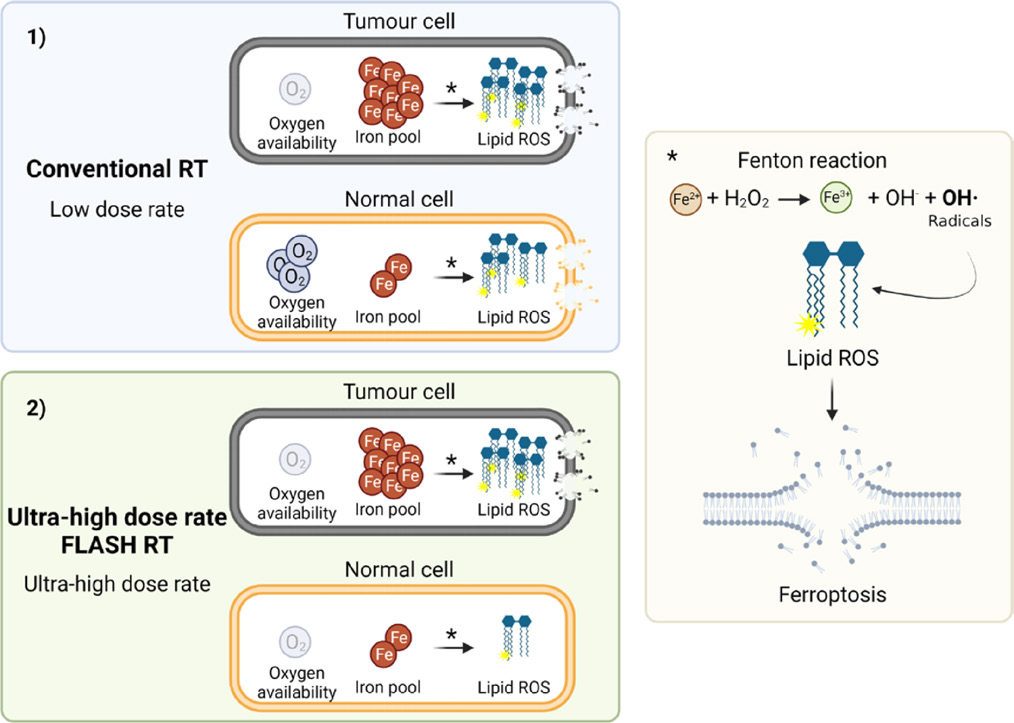
Hypothetical model of ferroptosis induction by ultra-high dose rate FLASH RT and conventional RT in normal ***vs*** tumour cells**.** In this theoretical model, we hypothesise that the protective effect found in normal tissue after ultra-high dose rate FLASH RT might be related to lipid peroxidation and ferroptosis induction. Briefly, compared to 1) conventional RT, 2) ultra-high dose rate FLASH RT induces oxygen depletion in normal cells,^8^ which protects them from ferroptosis mediated by lipid peroxidation. However, tumour cells already in hypoxic conditions would not have a differential effect from oxygen depletion between conventional and FLASH RT. Rather, due to a larger pool of labile iron in tumour cells compared to normal cells,^15^ they will experience a strong induction of lipid peroxidation after RT, which will lead to tumour cell killing. (*Fenton reaction, Created with BioRender.com, agreement number, *JY24HU5Q6T*).

To increase the therapeutic window of RT, tumour control needs to be achieved while protecting normal tissues. Great interest has been accumulated in both the ferroptosis and FLASH fields. Ferroptosis inducers have been proposed as an alternative method to enhance the radiosensitivity of tumour cells. On the other hand, FLASH RT has been shown to provide normal tissue protection. While recent studies have highlighted their potential in clinical settings, further progress is hampered by the lack of understanding of their underlying mechanisms. Based on previous and current studies, it can be suggested that the normal tissue protection after FLASH RT might be derived from the inverse dose rate effect on lipid peroxidation after radiation, which might translate into lower lipid peroxidation and, hence, less ferroptosis in normal tissues. Also, the lower capacity to process and remove labile iron in tumour tissues compared to normal may support the similar tumour cell killing effect observed after FLASH RT compared to conventional RT. This would imply that tumour cell death after FLASH radiation would be directly linked to ferroptosis (Figure 1). Although FLASH RT and ferroptosis seem to share a close link, there are still no studies connecting the two for therapeutic benefit. Therefore, it seems crucial to investigate whether ferroptosis plays a role in the normal tissue sparing derived from ultra-high dose rate FLASH RT and/or whether inducing ferroptosis can further promote tumour cell killing.

Until recently, studies on RT and lipid peroxidation were limited because the determination of lipid peroxidation through the measurement of malondialdehyde (MDA) and 4-HNE disrupts the end product of the reaction, yielding inaccurate results. In recent years, the development of fluorescent probes such as C11 BODIPY (581/591) and liperfluo ease the detection of lipid peroxidation in live cells and *in vivo* tumour tissues. Advances in methodology will lead to a better understanding of the mechanisms behind ferroptosis and RT and could be applied to study ferroptosis in the context of FLASH RT. Moreover, this could hopefully result in the clinical application of ferroptosis inducers. Finally, in conjunction with the current efforts to apply FLASH dose rates using proton and carbon-ion RT, it may also be valuable to determine the effect different radiation sources have on ferroptosis.
